# Keanu: a novel visualization tool to explore biodiversity in metagenomes

**DOI:** 10.1186/s12859-019-2629-4

**Published:** 2019-03-14

**Authors:** Adam Thrash, Mark Arick, Robyn A. Barbato, Robert M. Jones, Thomas A. Douglas, Julie Esdale, Edward J. Perkins, Natàlia Garcia-Reyero

**Affiliations:** 1 Institute for Genomics, Biocomputing & Biotechnology, Mississippi State University, Starkville, MS USA; 2 US Army Engineer Research and Development Center, Cold Regions Research and Engineering Laboratory, Hanover, NH USA; 3 US Army Engineer Research and Development Center, Cold Regions Research and Engineering Laboratory, Fort Wainwright, AK USA; 4 Center for the Environmental Management of Military Lands, Colorado State University, Fort Collins, CO USA; 5 US Army Engineer Research and Development Center, Environmental Laboratory, Vicksburg, MS USA

**Keywords:** Metagenomics, Visualization tool, Holocene, Alaska, Shotgun sequencing

## Abstract

**Background:**

One of the main challenges when analyzing complex metagenomics data is the fact that large amounts of information need to be presented in a comprehensive and easy-to-navigate way. In the process of analyzing FASTQ sequencing data, visualizing which organisms are present in the data can be useful, especially with metagenomics data or data suspected to be contaminated. Here, we describe the development and application of a command-line tool, Keanu, for visualizing and exploring sample content in metagenomics data. We developed Keanu as an interactive tool to make viewing complex data easier.

**Results:**

Keanu, a tool for exploring sequence content, helps a user to understand the presence and abundance of organisms in a sample by analyzing alignments against a database that contains taxonomy data and displaying them in an interactive web page. The content of a sample can be presented either as a collapsible tree, with node size indicating abundance, or as a bilevel partition graph, with arc size indicating abundance. Here, we illustrate how Keanu works by exploring shotgun metagenomics data from a sample collected from a bluff that contained paleosols and a krotovina in an alpine site in Ft. Greely, Alaska.

**Conclusions:**

Keanu provides a simple means by which researchers can explore and visualize species present in sequence data generated from complex communities and environments. Keanu is written in Python and is freely available at https://github.com/IGBB/keanu.

## Background

Metagenomics is the study of genetic material recovered from environmental samples. These samples provide information on the diversity and/or ecology of a specific environment. Metagenomic studies typically focus on the microbial sequences obtained from a shotgun sequencing dataset [1]. Characterization of the viral, bacterial, and eukaryotic components is critical to obtaining a holistic understanding of the diversity present in a specific sample [1]. The rapid development and improvement of sequencing platforms and tools allow researchers to better observe species richness and diversity in environmental samples. However, these advances have also resulted in the generation of a vast volume of data to be analyzed and interpreted. Analyzing large amounts of text data can be a tedious and difficult process. Metagenomics studies are not an exception. The datasets obtained are characterized by intrinsic multidimensionality and the presence of multiple levels of hierarchy and connectivity [2]. Visualization aids researchers in determining what further questions can be asked of the data and what additional analyses can be conducted. Interactivity adds further exploration and analysis options. The visualization of metagenomics data is an active area of research with new methods being developed every year. The visualization methods can be divided according to their visualization power. Some of the methods are intended to visually explore one single metagenome while others allow visualization of multiple metagenomes. The tools can also include different types of visualization such as pie charts, bubble charts, trees and dendrograms, box-plots, and self-organizing maps among others. For a detailed review of the different visualization methods and tools see [2].

Here, we developed a visualization tool, Keanu, that allows exploration of the species composition of a metagenome, including microorganisms, viruses, and eukaryotic species in order to investigate patterns of biodiversity in ancient soils in interior Alaska. The tool generates interactive web pages that can be opened locally and explored to determine how to proceed with further analysis of the samples based on their contents. This type of exploratory data analysis can often inform what types of additional questions can be asked of the data and whether more data should be collected [3]. While static images are useful for exploring small datasets, such images can be so large or so detailed, depending on the data, that exploring them becomes difficult. This interactive visualization shows the abundance of each taxon in descending order, from the root node to a species level using information from the National Center for Biotechnology Information (NCBI) taxonomy database. Users can explore particular subsections of the taxonomy without creating a chaotic, noisy visualization of the entire dataset.

Tools similar to Keanu have been developed. MetaSee [4], once available as a web service and standalone GUI application, was an interactive visualization tool capable of displaying metagenomic data in formats very similar to what Keanu uses. However, running MetaSee does not seem to be an easy task, since one would have to download the server source code and run it locally. Blobtools [5] and Metacoder [6] both create large static images. Blobtools is a python tool for taxonomic partitioning that creates blob plots. Metacoder [6] is an R package that creates heat trees. Both lack the interactive capability that drove the development of Keanu. Because Keanu is locally run, the user can maintain up-to-date databases rather than relying on a web service to maintain those databases or an organization to host a service, and Keanu’s interactivity aids in analyzing the output.

## Implementation

### Tool development

**Fig. 1:**
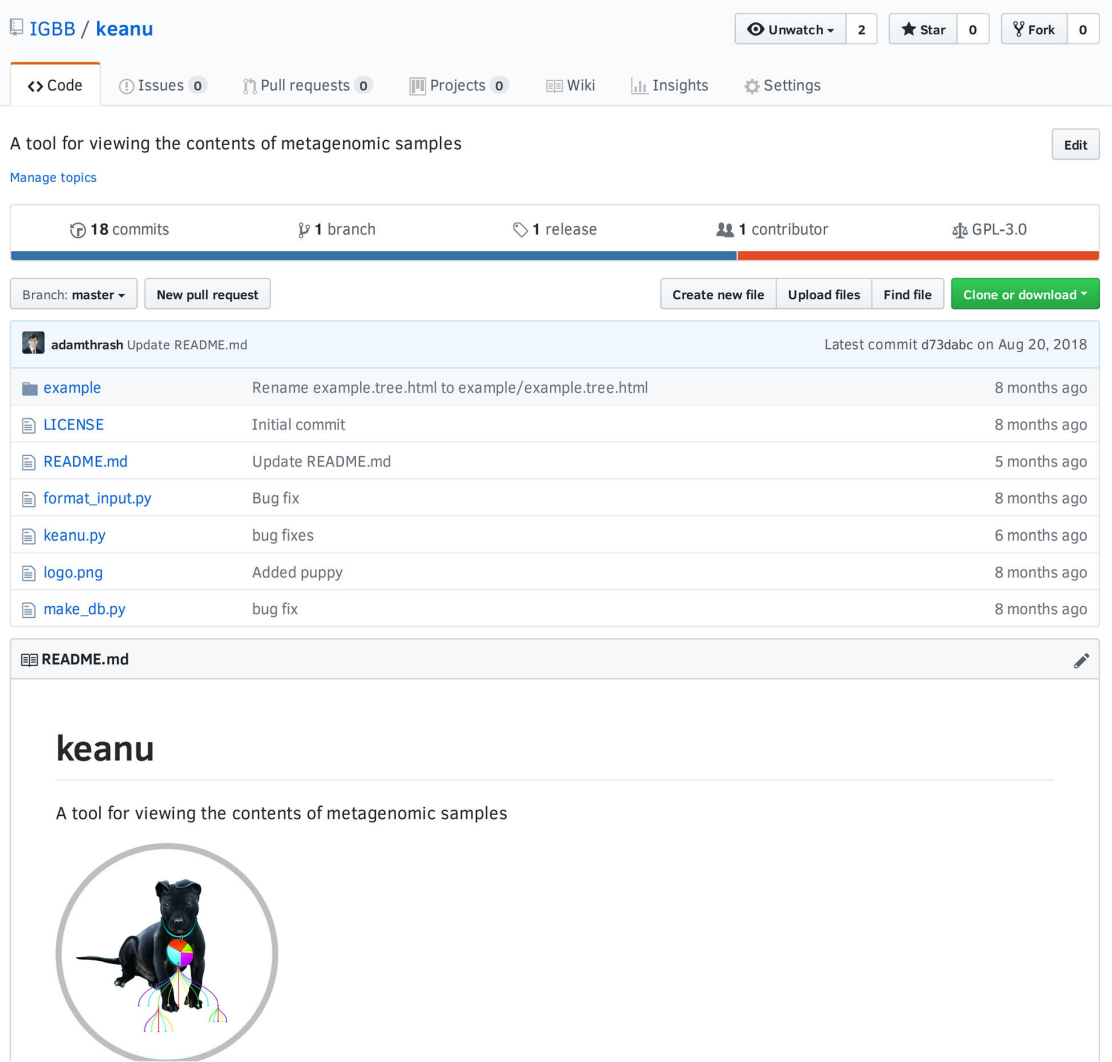
Keanu’s view at GitHub

Keanu is written in Python 3.5 and is available with an example dataset at https://github.com/IGBB/keanu (Fig. 1). It can be run on any system where Python is available, which includes Windows, macOS, and Linux. The program works by treating taxonomy as a tree. A root taxon in the NCBI taxonomy database forms the base of the tree and, as the tree branches out, classifications become more specific, from superkingdom to species. In the description of how Keanu works, “source” is used to refer to the classification immediately above the current classification, and “descendant” is used to refer to the classification immediately below the current classification. For example, species is the descendant of genus, and superkingdom is the source of kingdom.

Two classes of data are used in Keanu’s implementation to store the data pulled from NCBI. A Taxon object, referred to as a “node,” stores all data associated with an individual taxon. This data includes an NCBI-assigned unique identifying number, a name, a rank, a list of descendants, a source, and the number of hits that are assigned to the taxon. A Graph object, referred to as a “tree,” is used to organize how individual nodes are linked and to make traversing the links between nodes easier.

### Preparation of data

Keanu generates interactive web pages as output that can be explored to determine how to proceed with further analysis of the samples based on their contents. This visualization shows the abundance of each taxon in descending order from the root node to the species level. Information about these taxa is pulled from NCBI’s taxonomy database. Only classifications in NCBI’s abbreviated lineage are displayed (i.e. superkingdom, kingdom, phylum, class, order, suborder, family, genus, species).

**Fig. 2:**
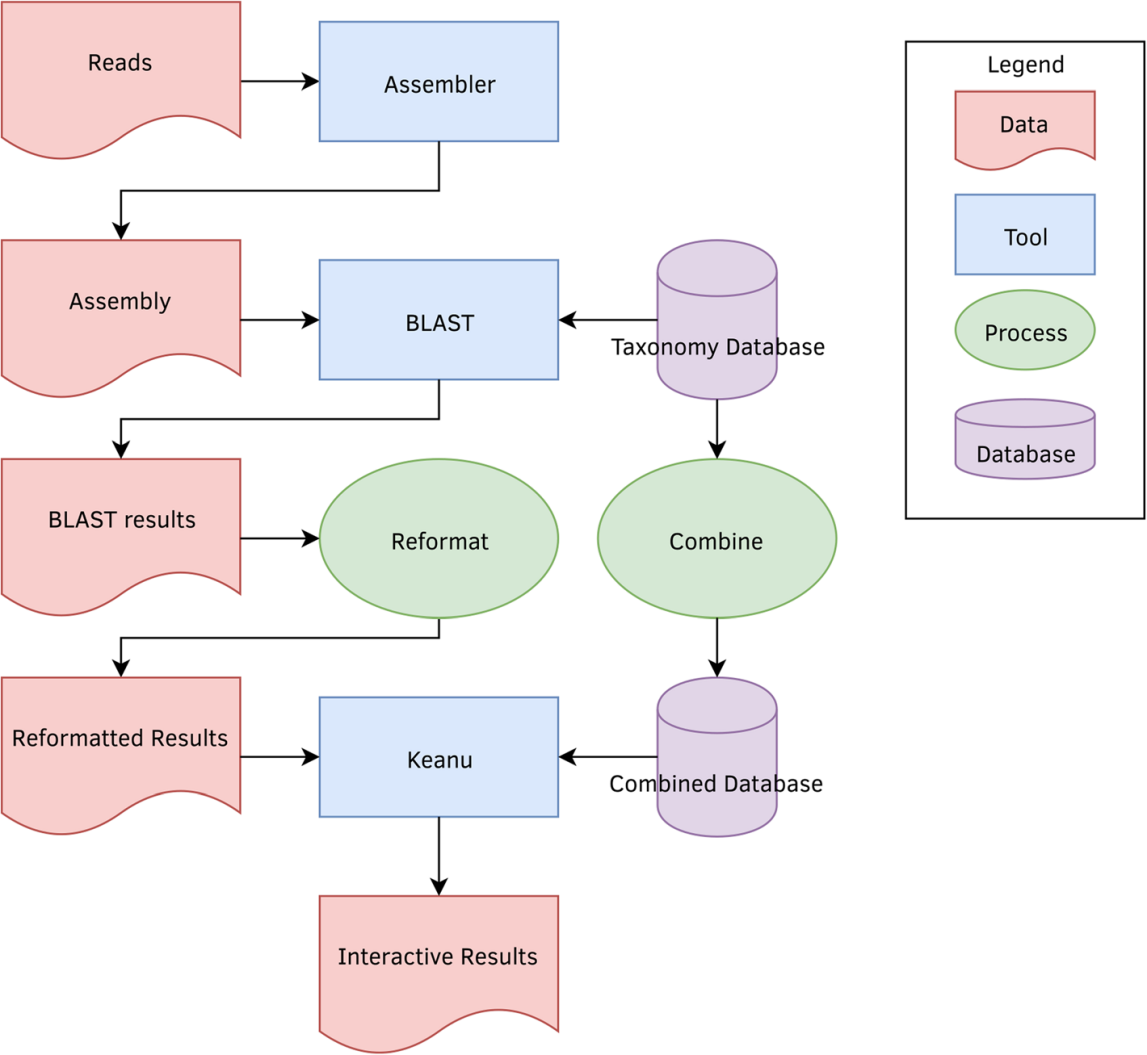
flow diagram of Keanu showing how data is piped into different tools or processes

The input files must be generated before Keanu itself is run (see Fig. 2). Raw FASTQ sequence data first needs to be trimmed to remove any adapter sequences or low-quality data. For the purposes of developing Keanu, sequence reads from the soil sample were trimmed using Trimmomatic 0.32 [7]. As an option, a user can reduce the number of reads (and files) used for alignment to known sequences by assembling sequence reads using an assembly program. Here, the trimmed sample data were assembled using ABySS 1.9.0 [8] and k-mers of length 70, 75, 80, and 85. Assemblies using 85-mers were selected because they had the best contiguity among the assemblies. The identity and taxonomy of sequence reads or assemblies were then determined by alignment to the NCBI Nucleotide (NT) [9] database using BLAST 2.2.30+ [10], though any aligner can be used so long as the results contain taxonomic data. These BLAST results can be filtered during the alignment stage using BLAST’s own filtering methods or using a scripting language of the user’s choice afterwards. Users may choose to do additional filtering with BLAST, such as excluding certain types of environmental data, before running Keanu. The resulting alignment file(s) were processed to produce a file containing a single query ID (a sequence read or contig from the assembly) and its associated taxon ID per line. Finally, this file was reformatted to contain all of the taxon data associated with a single query on one line, as well as the number of times that query-taxon pairing was seen in the file. A sample line might look like the example below.

QUERY_NAME taxon_id_1 [count], taxon_id_2 [count], taxon_id_3 [count]

In addition to the raw data from the samples, a taxonomy data file was downloaded from NCBI’s FTP website. The files containing data about the names and relationships of the taxa were combined into one file for easier parsing later, and the files containing information about merged and deleted taxa were combined as well. Combining these files reduces the number of files needed to run Keanu, and these databases can be stored for reproducibility more easily than keeping up with many individual files.

### Creating the tree

Keanu follows three steps to create its output. First, it creates the tree; then it populates the tree with the data supplied by the user; and finally, it produces an interactive web page for the user to explore based on publicly available D3 visualizations [11, 12]. To create the tree, Keanu reads the two files created earlier containing information about the taxonomy database downloaded from NCBI. As Keanu parses the file containing the taxonomy data, a node is created for each line in the file. All the information, except the number of times a node is encountered in the alignments, is present in the taxonomy database file. The second file containing the merged and deleted taxa data is then parsed, and a list of mappings from the original taxon identification number and the new merged identifying number is created. Any deleted taxa are deleted from the tree.

### Populating the tree

The data about taxon abundance must be added to each node in the graph. Keanu gets these data by reading the file that contains a line for each query and its associated data. If a query has multiple taxa associated with it, a single definitive assignment cannot be made because the alignment of the sequence itself was ambiguous. Instead, the most specific classification shared by all the taxa associated with a query is assigned a hit. For example, a query with hits to a wide variety of birds will have a hit assigned to *Aves* if *Aves* is the most specific classification shared by those hits. If a query has multiple taxa that share the same genus, then the hit is assigned to the shared genus. Rather than make ambiguous assignments, Keanu makes lower resolution but accurate assignments. Keanu determines the most specific shared classification by working backwards from the taxa associated with a query to the root of the tree and finding where the first branching into different classifications occurs. Once a hit is assigned, that hit is propagated upwards through the source of each node until the propagation reaches the root.

### Traversing the tree

Having populated the tree with numbers of hits to each taxon, Keanu traverses the tree using a recursive depth-first search. When Keanu first encounters a node, it visits the first descendent of that node, always selecting the first descendent until it reaches a node with no descendents. When a node with no descendants is discovered, Keanu backtracks to the previous node and visits the next descendant in the list. While there are easier ways to traverse the tree, this method allows Keanu to more easily create JSON-formatted data about its path through the tree. This format is for creating the visualization later. Nodes that are not in the abbreviated lineage are not included in the JSON output, and nodes without hits are not visited. When Keanu is done traversing the tree, the JSON-formatted data is cleaned up to be valid JSON data. Rather than providing the user with a JSON file for use with a separate HTML file, Keanu produces an HTML file with the JSON data included in the file for easy distribution to users. The analysis takes about 30 min to run, although time will depend on the processor specifications, computer memory, and size of the input data to Keanu.

### Soil sampling, DNA extraction and sequencing

The soil sample was collected aseptically using a sterile shovel from ancient soil in an archeological site located in Alaska in October 2015. The site was located on a 50 m high bluff overlooking the Delta River (63° 49′ 00”N and 14° 56′ 46”W). The bluff was composed of windblown silt and sand punctuated by a series of paleosols ranging from approximately 400 to 8000 calBP (calendar years before present). The sample was collected from an area where a krotovina was present. The krotovina itself, dated using radiocarbon methods, was 170 ± 30 calBP. Following collection, the soil sample was immediately placed onto dry ice and stored in the dark at 4 °C or − 80 °C for soil properties and DNA extraction, respectively. Genomic DNA was extracted from the soil using the PowerSoil® DNA isolation Kit (MoBio, San Diego, CA) and quantified using a Qubit 3.0 Fluorometer (Thermo Fisher Scientific, Waltham, MA). DNA was sent for sequencing to Global Biologics (Columbia, MO) and was run in the NexSeq Illumina platform (2 × 150 bp) as well as on the MiSeq Illumina Platform (2 × 250 bp). FASTQ files generated by the sequencers were then used for assembly and subsequent analysis by Keanu.

### Installing and running Keanu

Keanu can be downloaded from Github.com. Since Keanu is a Python script with accessory scripts for formatting databases and input, it does not need to be installed in a specific place in a computer.

Before running Keanu, some preparatory steps must be completed. Reads can be assembled using an assembler chosen by the user. Assembling reads reduces sequence duplication, which reduces the amount of data that must be aligned in later steps. Next, the reads or assembled sequences must be aligned to a database that contains taxonomy information from NCBI’s taxonomy database. If the user is using BLAST, then the output should be formatted in output format 6 (a tabular format easily parsed by other programs) and taxon information should be included with the staxids argument. If BLAST has already been run, perhaps for another part of the analysis, the taxonomy information can be extracted from the database and added to the BLAST results with a combination of UNIX commands. More detail is available in the README file on GitHub.

By design, no database is included with Keanu. Instead, Keanu includes a script to create an up-to-date database. The source of the database information is NCBI’s Taxonomy FTP site, where a user can download a compressed archive of the taxonomy information. Keanu’s make_db.py script can take the names file, the nodes file, the deleted nodes file, and the merged nodes file and combine them into a format used by Keanu.

After the database is created, the BLAST results or another aligner’s results can be parsed and reformatted. BLAST results by default have an entry for every match that a query has; Keanu requires that all information about a query be on a single line. The included reformatting script takes a file containing queries and taxonomy information from the alignment and reformats it for Keanu.

Finally, Keanu itself is run, taking as input the reformatted results from the previous step, the two databases created by the database creation step, and the user’s preference of the tree view or the bilevel partition graph view. Keanu produces a single, self-contained HTML file (web page) that can be sent to collaborators and viewed in a web browser.

## Results and discussion

**Fig. 3:**
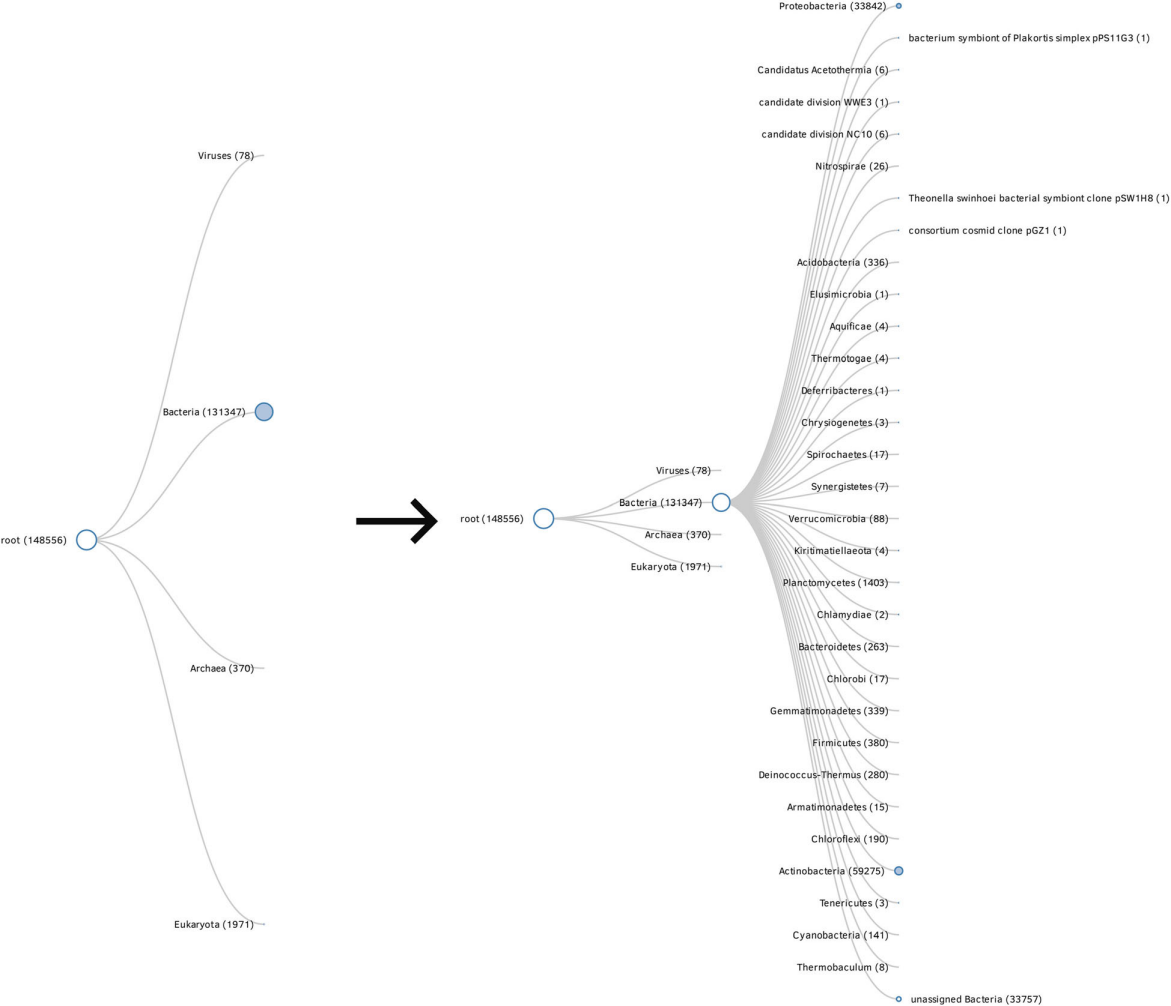
initial view using Keanu, showing first expansion of the nodes into sub-classifications, showing the name of the node and the number of hits to a node

Keanu produces an interactive collapsible tree or bilevel partition graph using publicly available code [11, 12]. This code has been slightly modified, and the modified version is included in Keanu. The collapsible tree initially displays the top categories when first opened in a browser. Each node’s name is displayed, as well as the number of hits associated with that taxon. A node’s size in this graph represents its size in comparison to the other nodes in the graph, though there is an upper limit on size to prevent nodes like the root node from filling the entirety of the graph. When a user clicks a node, the descendants of that node expand from it, and the graph reorganizes itself to better display the information (Fig. 3).

**Fig. 4:**
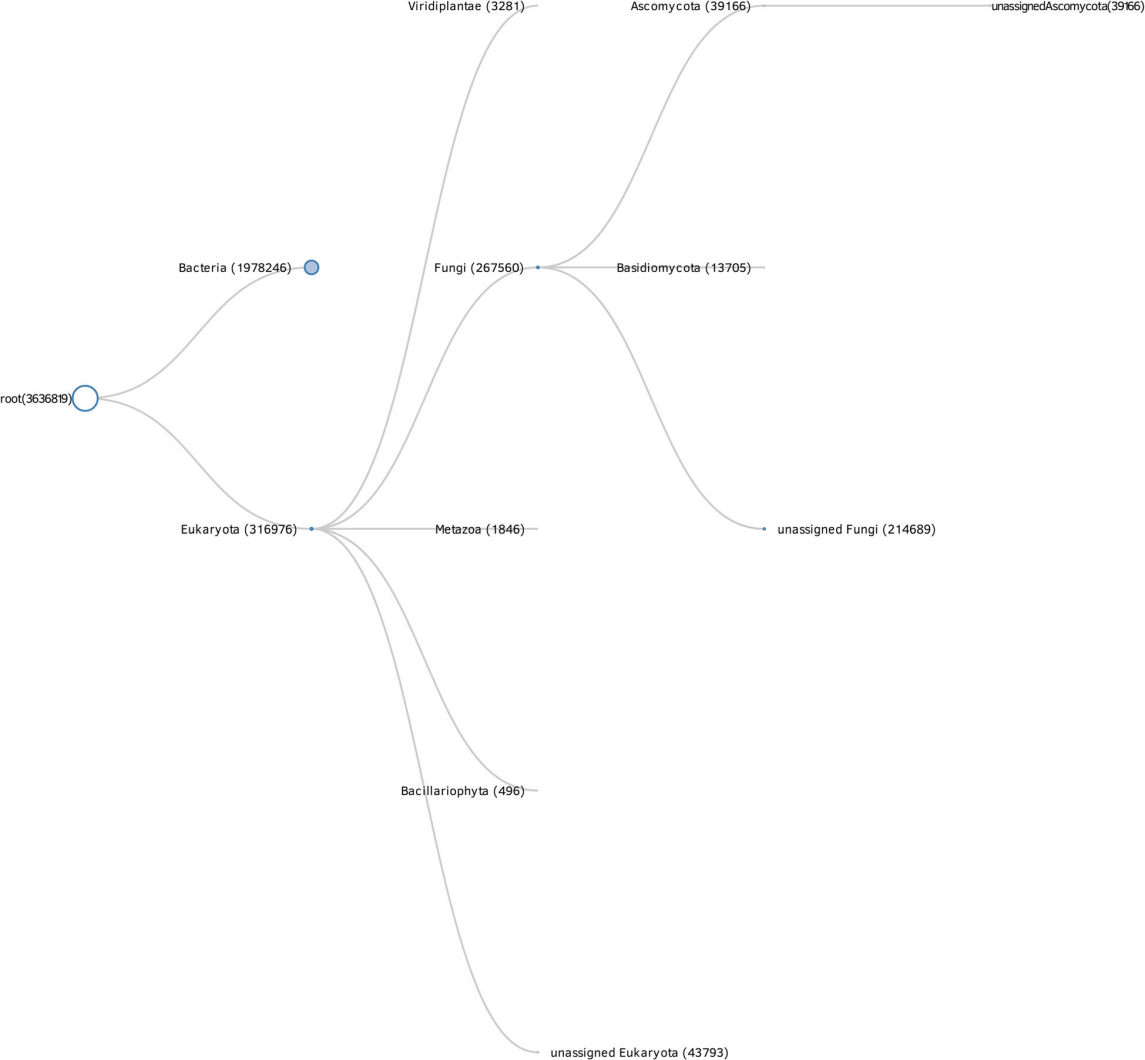
expanded view showing end of chain using a different, smaller dataset to show the end of a lineage

Nodes that can be expanded further are blue, and nodes that are expanded completely are empty. Clicking on either the name or the node itself expands or contracts the node. There is technically no limit on how a user chooses to expand and contract nodes, though the screen may eventually become difficult to read as nodes are forced off the canvas on which they are displayed. The final node in a chain has its name displayed to the right of the node rather than the left, making it easy for the user to determine when the chain has ended (Fig. 4).

**Fig. 5:**
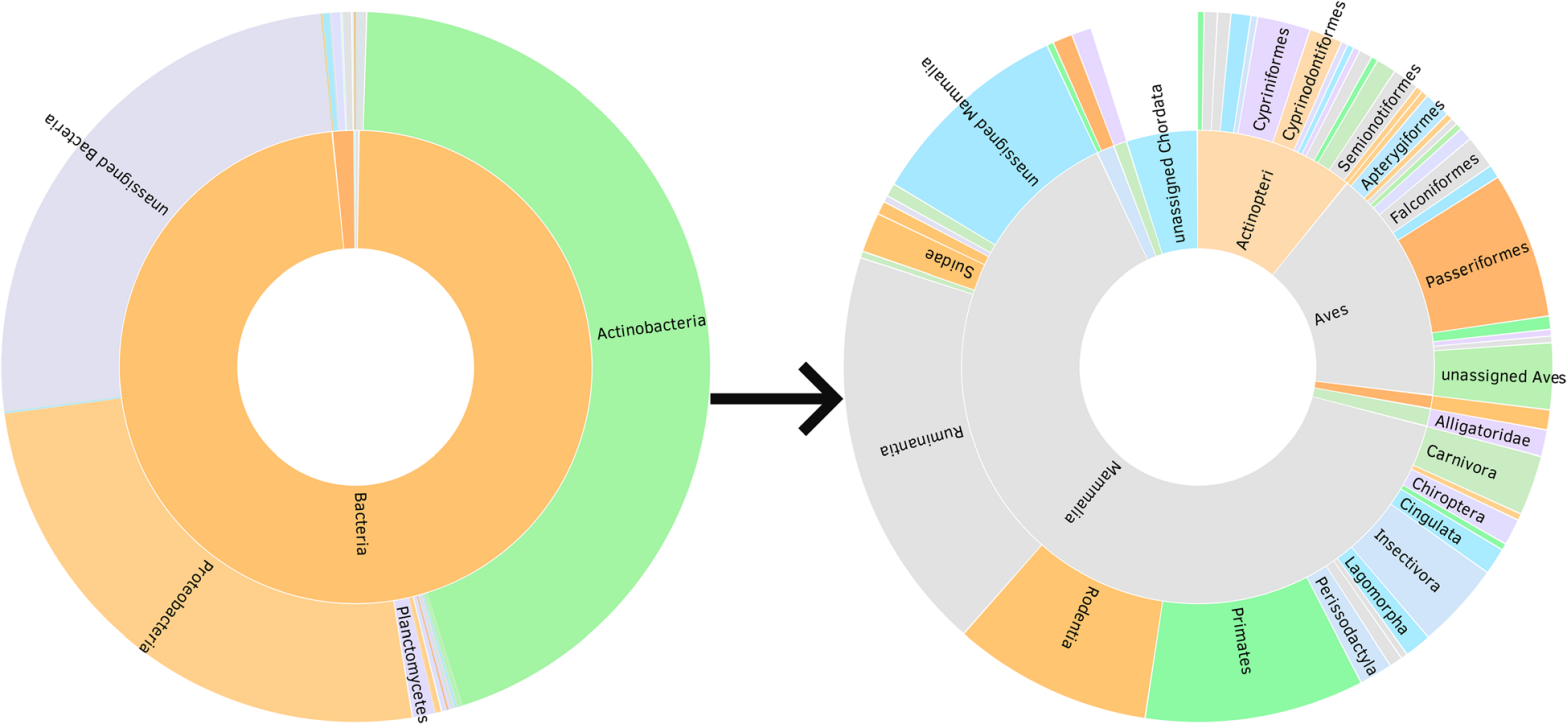
bilevel partition chart visualization of the metagenome. Keanu allows us to easily visualize the sample diversity and hypothesize on the potential species that lived in the nest. After clicking through several classifications, Keanu displays a bilevel partition chart visualization of the metagenome at the mammalian level

The data can also be visualized as bilevel partition charts, where clicking on a particular category will open a lower taxonomic level, and clicking in the middle of the chart will lead back to the previous taxonomic level (Fig. 5). These bilevel partition charts are particularly useful for examining the composition of a classification and determining the presence and abundance of sub-classifications. For example, determining what groups are present and their abundance within Chordata only requires looking at the circle for Chordata and seeing how it is divided into smaller pieces. Using the bilevel partition chart allows an easier comparison among the percentage of hits assigned to a classification at each level.

Several ideas guided the development of Keanu. First, Keanu was designed to be a command-line tool, with consideration towards running in high performance computing environments. Accordingly, Keanu lacks a guided user interface and instead has a simple command-line interface. Since access to the internet is not guaranteed in high performance computing environments, Keanu does not need an active internet connection to run, though a connection is needed for initial download of the databases from NCBI. Unlike hosted tools like MetaSee, Keanu does not rely on a third-party to host a service to run Keanu. As long as the source code is available, users can download and run Keanu. Second, Keanu’s visualizations should be interactive and easily shareable. Unlike Metacoder and Blobtools, Keanu generates interactive web pages rather than images. Interactivity allows users to focus only on the data they wish to view at the moment, rather than having all of the data spread across a large image. Keanu’s output is a web page, which can be published to the web by the user or sent to collaborators through nearly any messaging service. A web browser can be used to view results. Third, Keanu was designed to do one task – to output a visualization of the hits produced by aligning a metagenomic sample to a database with taxonomic visualization. Unlike Blobtools and Metacoder, Keanu is more a tool for quick and simple exploration and for guiding the user in determining any additional questions to ask of the data than for detailed and extensive analysis. Finally, Keanu was designed to be extensible. Nearly any visualization available from bl.ocks.org that uses the hierarchical example dataset (flare.json) can be converted to a visualization for Keanu, and any user skilled in D3 visualization could create entirely new visualization templates for Keanu.

### Visualization of Paleosol metagenome using Keanu

Interior Alaska has a unique Holocene sedimentary record due to historical and ongoing deposition of glacial-derived silt, or loess, across the landscape [13]. While katabatic winds produced substantial loess deposition in Central Alaska, periods of stability allowed the formation of paleosol sequences over the last 14,000 years [13, 14]. Thus, paleosols have developed sequentially through time and represent a unique *snapshot* of the conditions, including paleoclimate and biodiversity, from the time they were formed. Krotovinas, or the burrows of small mammals, can provide a very valuable archive of the biological activities of soil [15]. We used Keanu to visualize the metagenomics data from a soil sample obtained from a krotovina found within a bluff in Alaska. The sample sequencing resulted in a total of 20,984,020 reads.

The goal was to understand overall biodiversity in the sample, as well as to elucidate the species that might have been responsible for the krotovina found in the sample. The rationale was that the species that made and/or lived in the nest would have a higher percentage of DNA hits identified in the shotgun sequencing analysis. Data exploration using Keanu suggested that the nest might be related to the order *Rodentia* (Fig. 5). Moving further into the visualization, data suggested the nest could be related to the *Mus* species. Nevertheless, according to the fauna present in the area at the time, the krotovina belonged most likely to an Arctic ground squirrel (*Spermophilus parryii*) or a Hoary marmot (*Marmota caligata*, JE personal communication), which were not detected by our metagenomics analysis. It is worth noting that the fact that the database is heavily biased towards some species (i.e., mice, rat, human) could be responsible for a bias in the results as well. Ruminantia and primates had slightly more hits than rodents in this particular sample. Thus, expert knowledge had to be used to explore the species that potentially built the krotovina. Ideally, a more targeted sequence capture approach would follow to better support identification of the species associated with it.

## Conclusion

Keanu was developed to aid in exploring metagenomics data in such a way that the results were simple to distribute to collaborators and useful for understanding what the data might contain. Attempting to read BLAST alignment data is neither simple nor useful – alignment files can be extremely large and gaining any useful information from them without an additional tool is difficult. Keanu’s interactive output can be generated once alignment results are obtained, and it can be used to inform continued analysis of the data. Keanu allowed us to explore the species present in a metagenome from a soil sample collected in Alaska and to generate some follow up hypotheses on species distribution. This suggests Keanu is not only a very useful tool to explore metagenomic samples, but could also be used in environmental awareness, such as environmental DNA (eDNA) approaches to visualize and identify the presence of some specific species, genus, order, or classes in environmental samples of interest.

## Availability and requirements

- **Project name: Keanu**
- **Project home page:** **https://github.com/IGBB/keanu**
- **Operating system(s): Platform Independent**
- **Programming language: Python 3.5**
- **Other requirements: requires Python 3**
- **License: GPL v3**
- **Any restriction to use by non-academics: not applicable**

## Abbreviations

calBP: Calendar years before present
NCBI: National Center for Biotechnology Information
